# Enhancing Field‐Like Efficiency Via Interface Engineering with Sub‐Atomic Layer Ta Insertion

**DOI:** 10.1002/advs.202412409

**Published:** 2024-12-16

**Authors:** Shuanghai Wang, Kun He, Caitao Li, Yongkang Xu, Xingze Dai, Taikun Wang, Yu Liu, Yao Li, Yongbing Xu, Liang He

**Affiliations:** ^1^ School of Electronic Science and Engineering Nanjing University Nanjing 210023 China; ^2^ National Key laboratory of Spintronics Nanjing University Suzhou 215163 China

**Keywords:** field‐like efficiency, interface engineering, interfacial rashba effect, perpendicular magnetic anisotropy, spin‐orbit torque

## Abstract

The prevailing research emphasis has been on reducing the critical switching current density (*J*_c_) by enhancing the damping‐like efficiency (*β*_DL_). However, recent studies have shown that the field‐like efficiency (*β*_FL_) can also play a major role in reducing *J*_c_. In this study, the central inversion asymmetry of Pt‐Co is significantly enhanced through interface engineering at the sub‐atomic layer of Ta, thereby inducing substantial alterations in the *β*_FL_ associated with the interface. The *β*_FL_ has shown a 123% increase, from −1.66 Oe/(MA cm^−^^2^) to ‐3.8 Oe/(MA cm^−^^2^). As a result, the multilayered Ta/Pt/Ta (0.3 nm insertion)/Co/Ta structure leads to a notable decrease in *J*_c_, exceeding a remarkable 90% compared to the simpler Ta/Pt/Co/Ta structure, ultimately achieving a significantly low value of 2.7 MA cm^−^^2^. These findings pave the way for the development of highly efficient and energy‐saving spin‐orbit torque (SOT)‐based spintronic devices, where further optimizations in interface engineering can unlock even greater potential in terms of reduced power consumption and enhanced performance.

## Graphical abstract

The central inversion asymmetry of Pt‐Co is significantly enhanced through interface engineering at the sub‐atomic layer of Ta, thereby inducing substantial alterations in the field‐like efficiency associated with the interface. Consequently, the multilayered Ta/Pt/Ta (0.3 nm insertion)/Co/Ta structure results in a notable decrease in *J*_c_, achieving a remarkably low value of 2.7 MA cm^−^^2^.

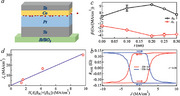

## 1 Introduction

With the appearance of current‐induced SOT, it has led to remarkable advancements in the design and operation of magnetic memory and logic devices.^[^ ^1^, ^2^, ^3^, ^4^, ^5^, ^6^ ^]^ The fundamental mechanisms of SOT, which originate from the bulk spin Hall effect (SHE) and/or the interfacial Rashba effect, are capable of generating two types of effective fields: damping‐like field (*H*_DL_) and field‐like field (*H*_FL_).^[^ ^7^, ^8^, ^9^ ^]^ These effective fields can manipulate the magnetization state of the adjacent ferromagnetic layers with perpendicular magnetic anisotropy (PMA).^[^ ^10^, ^11^ ^]^ Nevertheless, despite the immense potential demonstrated by SOT, its development is hindered by a significant challenge: the excessively high critical switching current density (*J*_c_), which typically falls within the range of 5–50 MA cm^−^^2^, significantly limiting its efficiency and feasibility in practical applications.^[^ ^12^, ^13^, ^14^, ^15^, ^16^, ^17^, ^18^, ^19^ ^]^

To mitigate the issue of high *J*_c_, a prevalent strategy is to enhance the spin Hall efficiency. This includes leveraging topological materials that exhibit strong spin‐orbit coupling, exemplified by (Bi, Sb_2_)Te_3_^[^ ^20^ ^]^ and Bi_0.9_Sb_0.1_,^[^ ^21^ ^]^ Bi_2_Se_3_,^[^ ^22^, ^23^, ^24^ ^]^ BiSb,^[^ ^24^, ^25^ ^]^ as well as incorporating doping impurities into heavy metal layers (Pt, W, or Ta), with materials such as MgO,^[^ ^26^ ^]^ TiO_2_,^[^ ^27^ ^]^ Ta,^[^ ^18^ ^]^ Cr,^[^ ^28^ ^]^ Au,^[^ ^29^ ^]^ Pd,^[^ ^30^ ^]^ Cu,^[^ ^31^ ^]^ and Al^[^ ^32^ ^]^ among the prominent choices, Similarly, some researchers have enhanced the spin Hall angle by utilizing different metallic phases, such as β‐W^[^ ^33^ ^]^ and β‐Ta.^[^ ^34^ ^]^ All the aforementioned aspects are achieved by enhancing the damping‐like efficiency (*β*_DL_), which is proportional to the spin Hall angle. On the other hand, recent reports have suggested that enhancing filed‐like efficiency (*β*_FL_) can also affect *J*_c_,^[^ ^35^, ^36^ ^]^ such as Mo/CoFeB/MgO heterostructure.^[^ ^37^ ^]^ Thus, finding new ways to influence *β*_FL_ is worth exploring to effectively lower *J*_c_.

In this study, sub‐atomic layers of Ta have been inserted into the Pt/Co interface during the growth process by sputtering. Anomalous Hall effect (AHE) measurements indicate that the sample possesses excellent PMA. The introduction of a sub‐atomic layer of Ta notably enhances the central inversion asymmetry of the interface, resulting in a significant 123% increase in *β*_FL_, from −1.66 Oe/(MA cm^−^^2^) to −3.8 Oe/(MA cm^−^^2^). At the same time, SOT switching measurements exhibited a remarkable reduction in *J*_c_ for the Ta/Pt/Ta (0.3 nm insertion)/Co/Ta sample, from 33.5 MA cm^−^^2^ at 1000 Oe to 2.7 MA cm^−^^2^ at 250 Oe. This discovery offers a novel approach for constructing low‐power SOT‐magnetic random‐access memory (SOT‐MRAM).

## 2 Results and Discussion

### 2.1 Sample Details

The multilayers of Ta (3 nm)/Pt (4 nm)/Ta (0–0.35 nm)/Co (0.8 nm)/Ta (2 nm) were deposited on Si/SiO_2_ substrates by magnetron sputtering with a base pressure of 3 × 10^−7^ Torr. The Ta layer at the bottom favors the PMA of Co. The top Ta layer functioned as a protective cover, preventing the oxidation of the Co film. The purity of Ta target is 99.95%, while Pt and Co are 99.99%. The sputtering gas used is 99.999% Ar for all the materials. For material deposition, the pressure is kept at 6 mTorr, and the direct current (DC) power is kept at 10 W. The deposition rates are 0.16, 0.2, and 0.14 Å s^−1^ for Ta, Pt, and Co layers, respectively. During deposition, the substrates were kept at room temperature.

**Figure 1:**
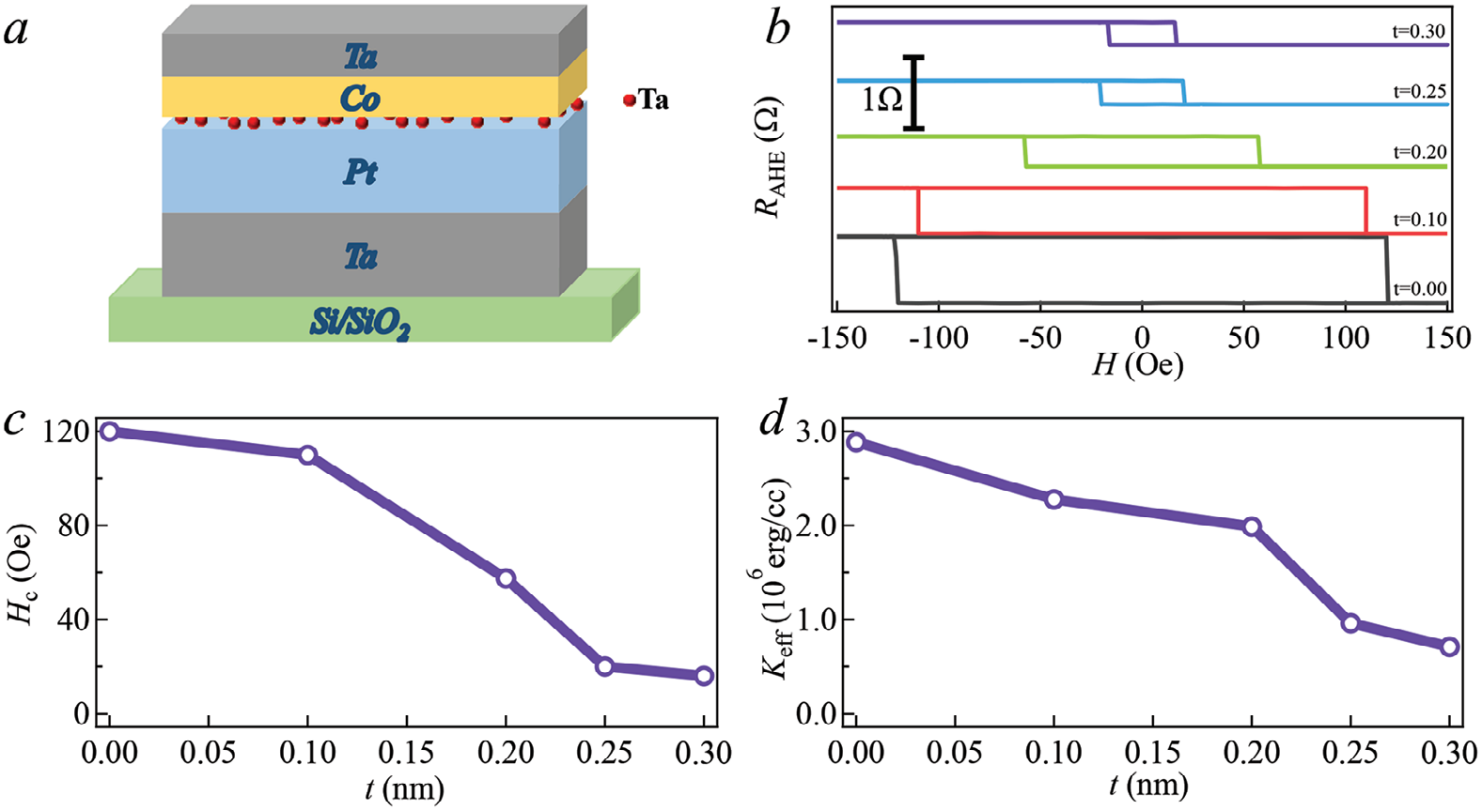
a) The structure of sample A series. b) The AHE loops of Sample A series (0–0.3 nm) exhibit out‐of‐plane easy axes. c) The *H*_c_ was extracted from AHE loops as a function of the Ta thickness. d) Changes in the *K*_eff_ values with varying *t* of the Ta insertion layer.

The structure of Sample A series is illustrated in **Figure** **1**a. Sample A0 is the basic structure without Ta insertion, and samples of A0.1 to A0.35 have the inserted Ta layer with the thickness of 0.1 to 0.35 nm, respectively. After deposition, the cross‐bar devices were fabricated using standard photolithography and ion beam etching processes (IBE). The width of the current channel is 10 µm. The AHE loops clearly demonstrate that the samples, ranging from A0 to A0.3, exhibit PMA, as illustrated in Figure 1b. On the other hand, sample A0.35 (*t* = 0.35) in Figure S1 (Supporting Information) displays a less vertical orientation, with a notable fraction of its magnetic moments already aligning in‐plane. The coercivity (*H*_c_) values, extracted from the AHE loops for all the samples, are plotted in Figure 1c, revealing a consistent decrease from 120 to 16 Oe as the Ta thickness gradually increases from 0 to 0.3 nm. The vibrating sample magnetometer measurement is conducted with the magnetic field oriented both parallel to the out‐of‐plane direction and to the in‐plane direction. To quantify the PMA, we use the effective magnetic anisotropy energy (*K*_eff_), which is defined as *K*_eff_ = *H*_k_ × *M*_s_/2, where, *H*_k_ is the effective PMA field, and *M*_s_ is the saturation magnetization. A positive *K*_eff_ value represents the PMA. The *K*_eff_ has been extracted for all the samples with different Ta insertion layers, as shown in Figure 1d. As the thickness of the Ta interlayer increases, the value of *K*_eff_ decreases.

### 2.2 The Measurement of the Second Harmonic in Ta (3)/Pt (4)/Ta (t)/Co (0.8)/Ta (2)

**Figure 2:**
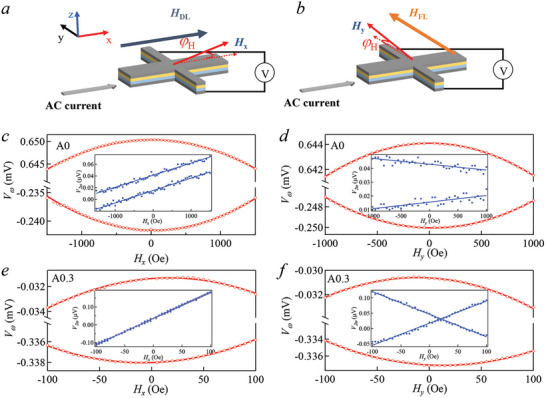
Shows schematic diagrams of the Hall device with a magnetic field applied in the x‐direction a) and y‐direction b) for conducting harmonic Hall measurements. Panels (c,d) display *V*_ω_ and *V*_2ω_, respectively, for sample A0. Panels (e,f) present *V*_ω_ and *V*_2ω_, respectively, for sample A0.3.

To probe the damping‐like torque and filed‐like torque from SOT‐induced spin current, the second harmonic method has been employed, as depicted in **Figure** **2**a,b. Refer to S2 for the determination of symbols for the *H*_DL_ and *H*_FL_ and field‐like torques in Pt/Ta/Co assist switching. To detect the *H*_DL_, a small external magnetic field (*H*_x_) is applied parallel to the current direction (*x*‐axis), with a slight inclination angle (*φ*_H_) to the *z*‐axis to prevent the formation of multiple domains.^18^ On the other hand, to probe the *H_FL_*, the external magnetic field (*H*_y_) is applied to align with the *y*‐axis with the same inclination angle. Alternating current (AC) of 0.5–2.5 mA with a frequency of 133 Hz in the *x*‐direction have been applied and simultaneously the first (*V*_ω_) and secondary (*V*_2ω_) harmonic signals are acquired through a lock‐in amplifier of OE1022D. Figure 2c–f shows *V*_ω_ and *V*_2ω_ as a function of magnetic fields *H*_x_ and *H*_y_, respectively, for samples A0 and A0.3, with an AC of 1 mA applied.

The longitudinal effective field (*H*_L/T_) induced by SOT can be quantitatively extracted, using the following formula:^[^ ^18^ ^]^

(1)

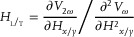




The *H*_DL_ or *H*_FL_ generated by the current can be acquired by the following formula:

(2) $${H_{\mathrm{DL}}}_{/\mathrm{FL}}=-2\frac{H_{L/T}+2\xi H_{L/T}}{1-4\xi^{2}}$$

**Figure 3:**
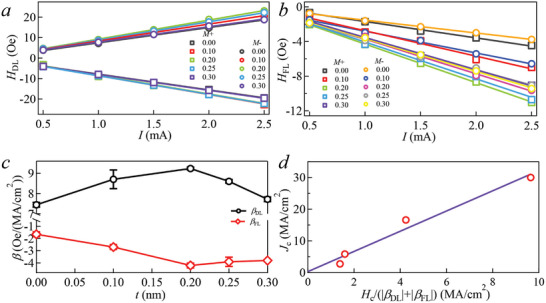
a) Current dependence of *H*_DL_ of sample A series. b) *H*_FL_ dependence on current for sample A series. c) The trend of *β*_DL_ and *β*_FL_ with different t. d) The linear relationship between the *J*_c_ and the *H*_c_/(|*β*_DL_| + |*β*_FL_|).

*ξ* is the ratio of planar Hall resistance to anomalous Hall resistance. In the Pt/Co system, planar Hall values are very small and are often neglected.^[^ ^18^ ^]^ So, *H*_DL/FL_ can be simplified as ‐2*H*_L/T_. **Figure** **3**a shows the plot of the extracted *H*_DL_ versus *I*_AC_ for all the samples, revealing a linear correlation with the applied current. Based on linear fitting, the *β*_DL_ is defined by the damping‐like efficient field per charge current density. As depicted in Figure 3c, the *β*_DL_ initially increases and then decreases, peaking at 9.2 Oe/(MA cm^−^^2^) of sample A0.2, representing a 24% enhancement compared to sample A0. The plot in Figure 3b depicts the extracted *H*_FL_ versus *I*_AC_ for all the samples, revealing a linear correlation with the applied current. The *β*_FL_ value for the A0 sample is −1.66 Oe/(MA cm^−^^2^), consistent with other reports.^[^ ^38^ ^]^ Notably, the field‐like torque efficiency *β*_FL_ exhibits a rise to −3.8 Oe/(MA cm^−^^2^) for sample A0.3, representing a 123% increase, as depicted in Figure 3c.

As depicted in Figure 3c, the insertion of Ta results in a non‐monotonic trend in field‐like torque efficiency with varying interlayer thickness. Initially, the efficiency increases due to the introduction of an extremely thin Ta layer (≤0.2 nm), which forms as islands. This island formation reduces the smoothness of the Pt/Co interface and enhances its asymmetry, thereby enhancing the Rashba effect. However, as the Ta interlayer thickness increases, it fills the gaps between these islands, enhancing the smoothness and reducing the asymmetry of the Pt/Co interface. Consequently, this leads to a weakening of the Rashba effect and a reduction in *β*_FL_.

Based on the domain‐wall depinning mode:^[^ ^39^, ^40^, ^41^ ^]^

(3) $$J_{c}=\frac{2e}{h}\mu_{0}M_{s}\left(t_{\mathrm{FM}}-t_{\mathrm{dead}}\right)\left(\frac{2}{\pi }\right)\left(\frac{H_{C}}{\beta_{D}}\right)$$

*J*_c_ is directly proportional to *H*_c_, suggesting that a reduction in *H*_c_ indeed leads to a decrease in *J*_c_. However, *J*_c_ is influenced not only by *H*_c_ but also by *β*_DL_ and *β*_FL_, which represent the driving forces for the switching of magnetization.^[^ ^35^, ^36^, ^37^ ^]^ Since when *H*_FL_ is anti‐parallel to *σ*, it assists the switch. Here we added *β*_FL_ into the equation. As shown in Figure 3d, the plot of *J*_c_ versus *H*_c_/(|*β*_DL_| + |*β*_FL_|) exhibits a clear linear relationship. This is strong evidence suggesting that the reduction of *J*_c_ is a combination effect of reducing *H*_c_ and enhancing |*β*_DL_| + |*β*_FL_|.

### 2.3 SOT‐Induced Magnetization Switching in Ta/Pt/Ta_t_/Co/Ta

To verify whether an increased *β*_FL_ can indeed lead to a reduction in *J*_c_, the schematic diagram has been illustrated for SOT‐induced magnetization switching on Ta/Pt/Ta_t_/Co/Ta Hall bar devices, as depicted in Figure S3 (Supporting Information). Where *M* denotes the magnetization direction of the ferromagnetic layer. When *I*_AC_ flows through the channel in the x‐direction, it generates a *H*_DL_ via the SHE. Simultaneously, the interfacial Rashba effect creates a *H*_FL_ that influences the magnetic moment.

**Figure 4:**
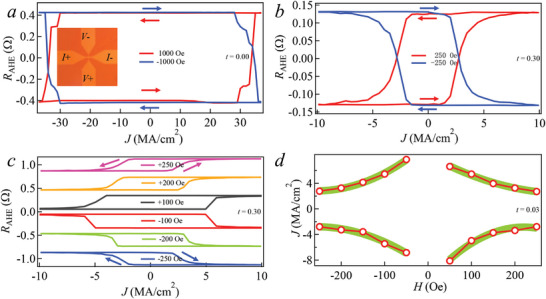
Magnetic and SOT properties for the A0 and A0.3. Current‐induced magnetization switching in the A0 a) under in‐plane magnetic field of *H*_x_ = ±1000 Oe and A0.3 b) under an in‐plane magnetic field of *H*_x_ = ±250 Oe. c) (Color online) Current‐induced magnetization switching in the A0.3, under various in‐plane magnetic fields *H*_x_ ranging from −250 to 250 Oe. d) Switching phase diagram of A0.3. The *J*_c_ decreases as the applied magnetic field increases.

The device configuration is illustrated in the inset of **Figure** **4**a. As depicted in Figure 4a, the SOT switching curve of sample A0 (without Ta insertion) exhibits a near‐perfect switch with the switching ratio, approaching 100%, when compared to the ΔR of AHE (as seen in Figure 1b). Notably, at a magnetic field of 1000 Oe, the *J*_c_ is 33.5 MA cm^−2^. In contrast, Figure 4b presents the SOT switching curves of sample A0.3 (with a Ta thickness of 0.3 nm) under applied magnetic fields of ±250 Oe. Remarkably, the *J*_c_ of A0.3 is only 2.7 MA cm^−2^, representing a substantial reduction of over 90% when compared to that of A0. The *J*_c_ value is relatively lower compared to 1.15 × 10^7^ A cm^−2^ for Pt(MgO) at 1500 Oe,^[^ ^26^ ^]^ 5.88 × 10^6^ A cm^−2^ for (Pt/Ta)_4_ at 1000 Oe,^[^ ^18^ ^]^ and 1.7 × 10^7^ A cm^−2^ for [Pt /Hf]_5_ at 3000 Oe.^[^ ^42^ ^]^ This suggests that the increased *β*_FL_ does contribute to the reduction of *J*_c_. Figure 4c demonstrates the switching loops of sample A0.3 under different in‐plane fields *H*_x_, parallel to the current. The SOT switching loops are counter‐clockwise under a positive magnetic field and clockwise under a negative magnetic field. Figure 4d exhibits the switching phase diagram of A0.3. The *J*_c_ of A0.3 decreases with the increase of the external magnetic field.

## 3 Conclusion

In summary, the insertion of a sub‐atomic layer of Ta significantly enhances the central inversion asymmetry of the Pt‐Co interface, leading to a particularly pronounced 123% increase in β_FL_, which is attributed to interfacial effects like the Rashba effect. Consequently, a remarkable reduction of over 90% in *J*_c_ was achieved, with the *J*_c_ value decreasing to 2.7 MA cm^−^^2^. Our findings highlight an efficacious approach to reducing the critical switching current via interlayer insertion, while offering tunability of *β*_FL_. This methodology holds significant potential for practical applications in SOT‐based spintronic devices.

## Conflict of Interest

The authors declare no conflict of interest.

## Supporting information



Supporting Information

## Data Availability

Research data are not shared.

## References

[1] F. Han , J. Zhang , F. Yang , B. Li , Y. He , G. Li , Y. Chen , Q. Jiang , Y. Huang , H. Zhang , J. Zhang , H. Yang , H. Liu , Q. Zhang , H. Wu , J. Chen , W. Zhao , X.‐L. Sheng , J. Sun , Y. Zhang , Nat. Commun. 2024, 15, 7299. 39181897 10.1038/s41467-024-51820-w PMC11344798

[2] S. C. Baek , K.‐W. Park , D.‐S. Kil , Y. Jang , J. Park , K.‐J. Lee , B.‐G. Park , Nat. Electron. 2018, 1, 398.

[3] L. Cao , Q. Chen , Y. Zhu , K. Tong , W. Li , J. Ma , M. Jalali , Z. Huang , J. Wu , Y. Zhai , ACS Appl. Mater. Interfaces. 2024, 16, 19764. 38577833 10.1021/acsami.4c00881

[4] X. Zhao , Y. Dong , W. Chen , X. Xie , L. Bai , Y. Chen , S. Kang , S. Yan , Y. Tian , Adv. Funct. Mater. 2021, 31, 2105359.

[5] Y. Li , N. Zhang , K. Wang , Sci. China Inf. Sci. 2022, 65, 122404.

[6] L. Yang , W. Li , C. Zuo , Y. Tao , F. Jin , H. Li , R. Tang , K. Dong , Appl. Phys. Lett. 2024, 125, 102402.

[7] J. Liu , X. Zha , Q. Lu , L. Liang , W. Wang , Z. Hu , Z. Guo , Z. Wang , M. Liu , ACS Appl. Mater. Interfaces 2024, 16, 49966. 39235948 10.1021/acsami.4c10495

[8] T. Z. Zhang , K. K. Meng , Y. Wu , J. K. Chen , X. G. Xu , Y. Jiang , Appl. Phys. Lett. 2024, 124, 202403.

[9] H. Ju , X. Zhao , W. Liu , Y. Song , L. Liu , J. Ma , Y. Li , J. Wu , Z. Zhang , ACS Appl. Mater. Interfaces. 2021, 13, 61742. 34905352 10.1021/acsami.1c17653

[10] K. Garello , I. M. Miron , C. O. Avci , F. Freimuth , Y. Mokrousov , S. Blügel , S. Auffret , O. Boulle , G. Gaudin , P. Gambardella , Nat. Nanotechnol. 2013, 8, 587. 23892985 10.1038/nnano.2013.145

[11] T.‐Y. Chen , W.‐B. Liao , T.‐Y. Chen , T.‐Y. Tsai , C.‐W. Peng , C.‐F. Pai , Appl. Phys. Lett. 2020, 116, 072405.

[12] K. Zheng , C. Cao , Y. Lu , J. Meng , J. Pan , Z. Zhao , Y. Xu , T. Shang , Q. Zhan , Appl. Phys. Lett. 2024, 124, 192408.

[13] J. W. Lee , Y.‐W. Oh , S.‐Y. Park , A. I. Figueroa , G. Van Der Laan , G. Go , K.‐J. Lee , B.‐G. Park , Phys. Rev. B 2017, 96, 064405.

[14] S. Łazarski , W. Skowroński , J. Kanak , Ł. Karwacki , S. Ziętek , K. Grochot , T. Stobiecki , F. Stobiecki , Phys. Rev. Appl. 2019, 12, 014006.

[15] J. Ryu , C. O. Avci , S. Karube , M. Kohda , G. S. D. Beach , J. Nitta , Appl. Phys. Lett. 2019, 114, 142402.

[16] X. Zhao , X. Zhang , H. Yang , W. Cai , Y. Zhao , Z. Wang , W. Zhao , Nanotechnology 2019, 30, 335707. 31018193 10.1088/1361-6528/ab1c02

[17] L. Zhang , X. Zhang , M. Wang , Z. Wang , W. Cai , K. Cao , D. Zhu , H. Yang , W. Zhao , Appl. Phys. Lett. 2018, 112, 142410.

[18] S. Wang , K. He , Y. Xu , Z. Li , J. Wang , C. Li , X. Dai , J. Du , Y.‐L. Wang , R. Liu , X. Lu , Y. Xu , L. He , Phys. Rev. Appl. 2024, 22, L021002.

[19] G. S. Li , Z. Z. Zhu , Z. Wang , J. T. Ke , P. J. Wang , L. Z. Bi , C. Q. Hu , Y. Zhang , J. W. Cai , J. Phys. Appl. Phys. 2024, 57, 305002.

[20] H. Wu , P. Zhang , P. Deng , Q. Lan , Q. Pan , S. A. Razavi , X. Che , L. Huang , B. Dai , K. Wong , X. Han , K. L. Wang , Phys. Rev. Lett. 2019, 123, 207205. 31809108 10.1103/PhysRevLett.123.207205

[21] N. H. D. Khang , Y. Ueda , P. N. Hai , Nat. Mater. 2018, 17, 808. 30061731 10.1038/s41563-018-0137-y

[22] M. Dc , R. Grassi , J.‐Y. Chen , M. Jamali , D. R. Hickey , D. Zhang , Z. Zhao , H. Li , P. Quarterman , Y. Lv , M. Li , A. Manchon , K. A. Mkhoyan , T. Low , J.‐P. Wang , Nat. Mater. 2018, 17, 800. 30061733 10.1038/s41563-018-0136-z

[23] A. R. Mellnik , J. S. Lee , A. Richardella , J. L. Grab , P. J. Mintun , M. H. Fischer , A. Vaezi , A. Manchon , E.‐A. Kim , N. Samarth , D. C. Ralph , Nature 2014, 511, 449. 25056062 10.1038/nature13534

[24] J. Han , A. Richardella , S. A. Siddiqui , J. Finley , N. Samarth , L. Liu , Phys. Rev. Lett. 2017, 119, 077702. 28949690 10.1103/PhysRevLett.119.077702

[25] Z. Chi , Y.‐C. Lau , X. Xu , T. Ohkubo , K. Hono , M. Hayashi , Sci. Adv,. 2020, 6, eaay2324. 32181344 10.1126/sciadv.aay2324 PMC7060068

[26] L. Zhu , L. Zhu , M. Sui , D. C. Ralph , R. A. Buhrman , Sci. Adv. 2019, 5, eaav8025. 31334348 10.1126/sciadv.aav8025 PMC6641942

[27] X. Xu , D. Zhang , B. Liu , H. Meng , J. Xu , Z. Zhong , X. Tang , H. Zhang , L. Jin , Adv. Sci. 2022, 9, 2105726. 10.1002/advs.202105726 PMC9165503 35393788

[28] C.‐Y. Hu , Y.‐F. Chiu , C.‐C. Tsai , C.‐C. Huang , K.‐H. Chen , C.‐W. Peng , C.‐M. Lee , M.‐Y. Song , Y.‐L. Huang , S.‐J. Lin , C.‐F. Pai , ACS Appl. Electron. Mater. 2022, 4, 1099.

[29] L. Zhu , D. C. Ralph , R. A. Buhrman , Phys. Rev. Appl. 2018, 10, 031001.

[30] L. Zhu , K. Sobotkiewich , X. Ma , X. Li , D. C. Ralph , R. A. Buhrman , Adv. Funct. Mater. 2019, 29, 1805822.

[31] C. Hu , C. Pai , Adv. Quantum. Technol. 2020, 3, 2000024.

[32] M.‐H. Nguyen , M. Zhao , D. C. Ralph , R. A. Buhrman , Appl. Phys. Lett. 2016, 108, 242407.

[33] C.‐F. Pai , L. Liu , Y. Li , H. W. Tseng , D. C. Ralph , R. A. Buhrman , Appl. Phys. Lett. 2012, 101, 122404.

[34] L. Liu , C.‐F. Pai , Y. Li , H. W. Tseng , D. C. Ralph , R. A. Buhrman , Science 2012, 336, 555. 22556245 10.1126/science.1218197

[35] Y. Zhuo , W. Cai , D. Zhu , H. Zhang , A. Du , K. Cao , J. Yin , Y. Huang , K. Shi , W. Zhao , Sci. China Phys. Mech. Astron. 2022, 65, 107511.

[36] D. Zhu , W. Zhao , Phys. Rev. Appl. 2020, 13, 044078.

[37] X. Wang , A. Meng , Y. Yao , F. Lin , Y. Bai , X. Ning , B. Li , Y. Zhang , T. Nie , S. Shi , W. Zhao , Nano Lett. 2024, 24, 6931. 38804717 10.1021/acs.nanolett.4c01100

[38] S. Emori , U. Bauer , S.‐M. Ahn , E. Martinez , G. S. D. Beach , Nat. Mater. 2013, 12, 611. 23770726 10.1038/nmat3675

[39] A. Thiaville , S. Rohart , É. Jué , V. Cros , A. Fert , EPL Europhys. Lett. 2012, 100, 57002.

[40] T.‐Y. Chen , C.‐T. Wu , H.‐W. Yen , C.‐F. Pai , Phys. Rev. B 2017, 96, 104434.

[41] L. Zhu , Adv. Mater. 2023, 35, 2300853. 10.1002/adma.202300853 37004142

[42] L. Zhu , L. Zhu , S. Shi , M. Sui , D. C. Ralph , R. A. Buhrman , Phys. Rev. Appl. 2019, 11, 061004.

