# Supplementary material for: Enhancing Field‐Like Efficiency Via Interface Engineering with Sub‐Atomic Layer Ta Insertion
Source: Adv Sci (Weinh). 2024 Dec 16;12(6):2412409. doi: 10.1002/advs.202412409 (PMC11809412; doi:10.1002/advs.202412409)
Supplement: Supplementary file 1 — Supporting Information [file ADVS-12-2412409-s001.docx]

**Supplementary Materials**

**S1. The P-MOKE loops of the samples**

The MOKE loops of the Ta (3nm)/Pt (4nm)/Ta (0.35nm)/Co (0.8nm)/Ta (2nm) sample indicate that a portion of its magnetic moments already exhibits IMA characteristics.

Figure S1. The MOKE loop of sample Ta (3nm)/Pt (4nm)/Ta (0.35nm)/Co (0.8nm)/Ta (2nm).

**S2** **The field-like torques in Pt/Ta/Co assist switching**.

First, the spin direction at the Pt/Co interface is along the negative y-axis, as illustrated in Figure S2, since the Pt has a positive spin Hall angle.^[41]^ Second, to determine the direction of *H*_FLT_ of our Pt/Co system, the Landau-Lifshitz-Gilbert (LLG) equation has been used. As reported in Communications Physics 1, 2 (2018), it accounts for both the damping-like and field-like components of spin-orbit torque, which is expressed as follows:

$\frac{d\hat{m}}{dt}$=-*γμ*_0_​$\hat{m}$×*H*_eff_​+*α*$\hat{m}$×$\frac{d\hat{m}}{dt}$−*γ*$\hat{m}$×(-*τ*_DL_$\hat{m}$​×$\hat{y}$​) − *γ*$\hat{m}$×(-*τ*_FL_​$\hat{y}$) (4)

where *τ*_DLT_​=*β*_DL_​( ℏJ)/2*eM_s_*​*d* and *τ*_FLT​_=*β*_FL_​( ℏJ)/2*eM_s_*​*d*. And *β*_FL_ (*β*_DL_) and *τ*_FLT_ (*τ*_DLT_ ) have the same sign. Then, the equivalent fields for the damping-like torque (DLT) and field-like torque (FLT) are defined as *H*_DL_​=-*τ*_DL_$\hat{m}$​×$\hat{y}$​​ and *H*_FL_=-*τ*_FL_​$\hat{y}$=-*β*_FL_​( ℏJ)/2*eM_s_*​*d*$\hat{y}$, respectively. Based on our measurement, the *β*_FL_ is negative. Then, *H*_FL_ is along the positive y-axis, as illustrated in Figure S2.

According to Sci. China-Phys. Mech. Astron. 65, 107511 (2022), when the direction of *H*_FL_ is opposite to that of the *σ*_spin_, it facilitates switching and thereby reduces the critical switching current density. The signs of *β*_FL_, *σ*_spin_, and *H*_FL_, as reported in various references, are presented in Table S1. And our work is consistent with those reported.

Table S1. The signs of *β*_FL_, *σ*_spin_, and *H*_FL_ in different references.

| Sample structure | *σ*_spin_ | *β*_FL_ | *H*_FL_ | promote/ inhibit switch | Ref. |
| --- | --- | --- | --- | --- | --- |
| Ta/CFB | **+^y^** | **+^y^** | **- ^y^** | promote | Sci. China-Phys. Mech. Astron. 65, 107511 (2022) |
| Pt/CoFe | **-** | **-** | **+** | promote | Nature Materials. 12, 611–616 (2013) |
| Pt/Ni/Py | **-** | **+** | **-** | inhibit | Physical Review B 110, 064419 (2024) |
| Pt/Co/Py | **-** | **-** | **+** | promote | Physical Review B 110, 064419 (2024) |
| Pt/Fe/Py | **-** | **-** | **+** | promote | Physical Review B 110, 064419 (2024) |
| Pt/Co | **-^y^** | **- ^y^** | **+ ^y^** | promote | This work |

Figure S2. Illustration of the directions of the current-induced effective field *H*_FL_ in Ta/Pt/Co/Ta.

**S3. Schematic diagram of SOT measurements**

M is the magnetization direction of the ferromagnetic layer. When I_AC_ flows through the channel in the x-direction, it generates a damping-like field (*H*_FL_) via the spin Hall effect. Simultaneously, the interfacial Rashba effect creates a field-like field (*H*_FL_) that influences the magnetic moment.

Figure S3. The schematic diagram for SOT-induced magnetization switching on Ta/Pt/Ta/Co/Ta Hall bar devices.
